# FreeGaze: A Framework for 3D Gaze Estimation Using Appearance Cues from a Facial Video

**DOI:** 10.3390/s23239604

**Published:** 2023-12-04

**Authors:** Shang Tian, Haiyan Tu, Ling He, Yue Ivan Wu, Xiujuan Zheng

**Affiliations:** 1 College of Electrical Engineering, Sichuan University, Chengdu 610065, China; 2 Key Laboratory of Information and Automation Technology of Sichuan Province, Sichuan University, Chengdu 610065, China; 3 College of Biomedical Engineering, Sichuan University, Chengdu 610065, China; 4 College of Computer Science, Sichuan University, Chengdu 610065, China

**Keywords:** gaze estimation, dual-branch CNN, improved normalization, eye features, face features

## Abstract

Gaze is a significant behavioral characteristic that can be used to reflect a person’s attention. In recent years, there has been a growing interest in estimating gaze from facial videos. However, gaze estimation remains a challenging problem due to variations in appearance and head poses. To address this, a framework for 3D gaze estimation using appearance cues is developed in this study. The framework begins with an end-to-end approach to detect facial landmarks. Subsequently, we employ a normalization method and improve the normalization method using orthogonal matrices and conduct comparative experiments to prove that the improved normalization method has a higher accuracy and a lower computational time in gaze estimation. Finally, we introduce a dual-branch convolutional neural network, named FG-Net, which processes the normalized images and extracts eye and face features through two branches. The extracted multi-features are then integrated and input into a fully connected layer to estimate the 3D gaze vectors. To evaluate the performance of our approach, we conduct ten-fold cross-validation experiments on two public datasets, namely MPIIGaze and EyeDiap, achieving remarkable accuracies of 3.11° and 2.75°, respectively. The results demonstrate the high effectiveness of our proposed framework, showcasing its state-of-the-art performance in 3D gaze estimation.

## 1. Introduction

Gaze is a useful behavioral characteristic for reflecting a person’s attention and has found applications in various fields, such as human–computer interaction [1,2], action recognition [3], healthcare monitoring [4,5], and reading analysis [6]. As a result, accurate gaze estimation has become a topic of increasing interest in recent years, highlighting the importance of estimating gaze direction with precision.

Gaze estimation refers to estimating the direction or landing point on a specific plane. It can be roughly divided into two categories: model-based and appearance-based. Most of the existing commercial eye trackers rely on model-based methods for gaze estimation, which establish a three-dimensional geometric model for gaze or fixation point estimation with the pupil center corneal reflection method [7]. In order to calculate the required parameters of the model, these model-based methods generally need to detect feature edges clearly, such as near-infrared corneal reflection [8,9], iris contour [10,11], and pupil center [12]. Therefore, they mostly rely on specialized equipment, such as near-infrared light, depth cameras, RGB-D cameras, etc. Although these model-based methods can provide high accuracy, they still have several limitations, including (1) high cost of equipment; (2) complex models and calibration procedures; and (3) strong restrictions on head movements. These factors limit their application scenarios, and they can only be used indoors or in laboratory environments.

Appearance-based methods learn gaze mapping directly from appearances of the face or eyes in facial videos. Compared to model-based methods, appearance-based methods only require a single ordinary RGB camera for data collection and can work without explicit eye feature detection. Hence, the appearance-based methods for gaze estimation have a wider range of application scenarios, not only indoors but also outdoors, and have less restriction on head movements. Unfortunately, despite these advantages, there are still many challenges with appearance-based methods, such as different illuminations, various head poses, and appearance differences among different individuals. These factors greatly increase the complexity of the data, thus making it difficult to learn the mapping from eye or face appearances; as a consequence, the result of appearance-based methods for gaze estimation is still not accurate enough. Some researchers have tried to increase the accuracy of gaze estimation by using traditional machine learning methods, such as Support Vector Regression [13], k-Nearest Neighbors [13,14], and Random Forests [14]. However, due to the limited learning and expressive capabilities of traditional machine learning methods, they cannot achieve satisfactory accuracy in gaze estimation.

In recent years, deep learning methods have demonstrated excellent performance in classification, recognition, regression, and other visual tasks [15]. As a consequence, gaze estimation using deep learning methods has attracted more attention, especially convolutional neural networks (CNNs). For example, some useful networks, such as AlexNet [16] and VGG [17], have been proven to be effective in gaze estimation.

With the development of appearance-based gaze estimation, the datasets containing various illuminations, head poses, and appearances, such as MPIIGaze [18] and EyeDiap [19], have been published for gaze estimation via facial image sequences or videos. Intuitively, eye images carry the richest information of the gaze direction, and are first considered to predict gaze direction. As early as 2015, Zhang et al. used LeNet to estimate gaze from the monocular image and significantly increased the accuracy of gaze estimation compared with conventional machine learning methods [18]. Park et al. proposed learning an intermediate graphical representation of the eye, which is then used by a very lightweight DenseNet to estimate gaze direction [20]. Lian et al. proposed a shared CNN and used eye images captured by multiple cameras to estimate gaze direction [21]. Liu et al. proposed directly training a differential network to predict the gaze difference between two eyes’ gazes of one subject [22]. Huang et al. proposed a differential residual model (DRNet) combined with a new loss function for gaze estimation using the difference information of two eye images [23]. Yu et al. proposed an unsupervised representation learning for gaze estimation using eye images, addressing the issue of difficulty in collecting large and diverse data [24].

In addition to eye features, the face features, such as head pose and facial appearances, can also influence the performance of gaze estimation. Other researchers have considered these factors and conducted experiments to verify facial features for obtaining gaze estimations. Zhang et al. encoded the full face image using AlexNet with spatial weights and improved the accuracy of gaze estimation [16]. Ren et al. proposed a bilinear pooling-based attention CNN to extract full face features for accurate gaze estimation [25]. Palmero et al. combined face image, eyes region, and face landmarks as individual streams in a VGG-like network to estimate gaze in still images, and they utilized sequence information to predict the gaze direction of the last frame [17]. Gu et al. proposed a differential gaze estimation method by combining eye images and normalized head pose information [26]. Krafka et al. proposed iTracker, a CNN for gaze estimation, which takes left and right eye images, a facial image, as well as a face grid as inputs for 2D gaze estimation on mobile devices [27]. Zhou et al. proposed an improved iTracker, which took face images and eye images as inputs for gaze estimation of a single frame. For videos, they employed a many-to-one BiLSTM to fit the time information between frames to predict the gaze of the last frame [28]. Kellnhofer et al. proposed the Gaze360 model, a combined model of CNN and LSTM that uses multiple face images as input, to predict gaze while outputting an estimate of gaze uncertainty [29]. Chen et al. proposed a gaze decomposition method that took face and eye images as inputs to the network for gaze estimation [30]. Li et al. proposed the static transformer with a temporal differential network (STTDN) for gaze estimation using face and eye images as input [31]. Overall, using more facial information for gaze estimation results in higher accuracy compared to only using eye images.

The eye features include important appearances, such as location of the iris for gaze estimation. The face features consist of the orientation of faces with respect to gaze direction. Therefore, it is reasonable to combine eye features and face features to predict gaze direction. However, most researchers have not studied whether using facial landmarks from different facial regions for normalization has an impact on the accuracy of gaze estimation. Also, they have not studied the difference between the improved normalization method and the original normalization method and their impact on gaze estimation accuracy.

In this study, we aim to study the contributions of eye and face appearances to gaze estimation and study the impact of using facial landmarks in different facial regions for data normalization on the accuracy of gaze estimation. Moreover, we have improved the normalization method so that the angle between the ground truth of gaze vector and the prediction of gaze vector can be calculated in the normalized coordinate system without the need to convert to the original camera coordinate system. Table 1 shows the comparison of our method with other state-of-the-art methods in terms of features (eye and face) and advantages, in which y denotes yes and n denotes no.

The contributions of this paper are concluded as:

1. We develop a framework, named FreeGaze, for appearance-based 3D gaze estimation from facial videos and study the contributions of face and eye features.
2. We improve the normalization method using orthogonal matrices, proving that the improved normalization method has a higher accuracy and a lower computational time in gaze estimation.
3. We propose a dual-branch CNN, which combines face and eye appearances for gaze estimation, and evaluate the contribution of both face and eye features separately.
4. We study the effect of facial landmarks in different facial regions for normalization on gaze estimation accuracy.

## 2. Method

**Figure 1:**
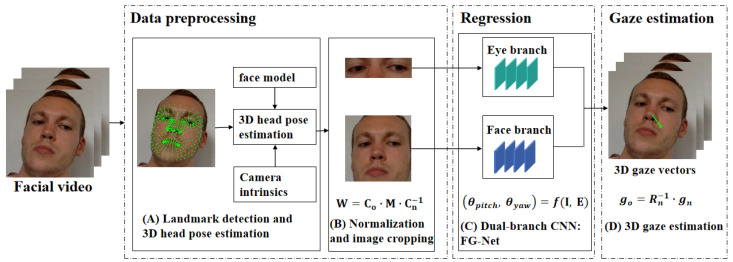
The workflow of FreeGaze for 3D gaze estimation. It mainly consists of four steps: (**A**) landmark detection and 3D head pose estimation, which is the basis of normalization; (**B**) normalization and image cropping. In this step, the normalized eye and face images are cropped as the inputs for deep learning; (**C**) a dual-branch CNN, named FG-Net. The eye branch is used for eye feature extraction, and the face branch is used for face feature extraction. Gaze angle vector is estimated through network regression; (**D**) the 3D gaze vector is computed from gaze angle vector and converted to original camera coordinate system, and the green arrow represents the gaze vector in a frame.

Figure 1 shows the workflow of FreeGaze for appearance-based 3D gaze estimation. There are three main steps. We first detect the face and locate facial landmarks in a frame of a facial video. Then, we estimate 3D head poses of the faces and apply space normalization method [14] to crop and warp face images to the normalized space. Finally, we use the normalized data as inputs for the dual-branch CNN to estimate the gaze in this frame. The details of the three steps are described as follows.

### 2.1. Facial Landmarks Detection and 3D Head Pose Estimation

We first detect the face in the frame of a facial video using Bazarevsky’s BlazeFace method [32]. Then, we employ FaceMesh method proposed by Grishchenko et al. [33] to obtain facial landmarks.

The FaceMesh method detects 468 facial landmarks and outputs their 3D coordinates, in which x- and y-coordinates indicate normalized pixel coordinates in [0, 1], and z-coordinates are depth data. Next, we obtain the definition of the canonical face model [33]. The canonical face model consists of 3D positions of 468 facial landmarks. Considering the x- and y-coordinates, we compute the head pose $R_{h}$ by estimating the initial solution using EPnP algorithm [34] with the canonical face model and the detected x- and y- coordinates, and further refining the head pose vector $r_{h}$ via non-linear optimization. Finally, we convert the head pose vector $r_{h}$ into the head pose matrix $R_{h}$ using the Rodrigues formula. The head pose matrix $R_{h}$ is an orthogonal matrix.

### 2.2. Normalization

The frames in the facial videos contain a lot of redundant information, such as background information. Moreover, the original head pose contains 6 degrees of freedom with respect to the camera coordinate system, and in this case the gaze estimator has to handle appearances in the 6D space. To reduce the degrees of freedom and minimize the impact of redundant information on gaze estimation, a perspective transformation is applied to convert the original image into a specific normalized space. The application of perspective transformation greatly reduces the prediction difficulty and the number of model parameters required, leading to a more streamlined and efficient performance of gaze estimation. The normalization method used in this paper is inherited from the previous study [14].

After applying perspective transformation, the normalized image would meet three conditions. First, the z-axis of the virtual camera in the normalized space points towards the reference point and the center of the face is located at the center of the normalized image. Second, the x-axis of the head coordinate system is parallel to the x-axis of the virtual camera coordinate system; in other words, the line connecting the two eyes is a straight line. Third, the virtual camera in the normalized space is located at a fixed distance $d_{n}$ from the reference point (the face center) and the normalized images have the same size.

For the first and second conditions, the rotation matrix $R_{n}$ between the camera coordinate system and the virtual camera coordinate system can be described as follows. Assuming that the rotation matrix from the camera coordinate system to the head coordinate system $R_{h}$ is as Equation (1):

(1) $$R_{h}=\left\{x_{h},y_{h},z_{h}\right\}$$

where $x_{h}$, $y_{h}$, $z_{h}$ represent the x-axis, y-axis, and z-axis of the head pose in the camera coordinate system. Assuming that the coordinate of the reference point in the camera coordinate system is m, to make the z-axis of the virtual camera point towards the reference point, its z-axis $z_{n}$ has to be

(2) $$z_{n}={\frac{m}{∥m∥}}_{2}$$

To satisfy the second condition, the y-axis of the normalized camera $y_{n}$ has to be defined as Equation (3):

(3) $$y_{n}=z_{n}\times x_{h}$$

then, the x-axis $x_{n}$ of the virtual camera coordinate system is

(4) $$x_{n}=y_{n}\times z_{n}$$

so we obtain the rotation matrix $R_{n}$ as Equation (5):

(5) $$R_{n}=\left\{x_{n},y_{n},z_{n}\right\}$$

The scaling matrix S can be defined as Equation (6) so that the virtual camera in the normalized space is located at a fixed distance $d_{n}$ from the reference point:

(6) $$S=diag\left\{1,1,{\frac{d_{n}}{∥m∥}}_{2}\right\}$$

therefore, we obtain the transformation matrix:

(7) $$M=S\cdot R_{n}$$

afterward, we define the intrinsic matrix $C_{n}$ of the virtual camera, which allows us to generate the desired size of normalized images. Then, we implement the perspective transformation by the warp perspective matrix presented in Equation (8):

(8) $$W=C_{o}\cdot M\cdot C_{n}^{-1}$$

where $C_{o}$ is the intrinsic matrix of the original camera.

In addition to images, we also have to convert gaze vector into the virtual camera coordinate system using Equation (9):

(9) $$g_{n}=R_{n}\cdot g_{o}$$

where $g_{o}$ is the original gaze vector and $g_{n}$ is the transformed gaze vector in the virtual camera coordinate system. The $g_{n}$ can be further represented in a 2D angle space $\left(\theta_{pitch},\theta_{yaw}\right)$ in order to reduce the complexity of regression, where $\theta_{pitch}$ and $\theta_{yaw}$, respectively, denote the vertical and horizontal direction angles. Let $g_{n}$ be

(10) $$g_{n}={\left(x_{gn},y_{gn},z_{gn}\right)}^{T}$$

where $x_{gn},y_{gn},z_{gn}$, respectively, represent the x coordinate, y coordinate, and z coordinate of gaze vector $g_{n}$ in the virtual camera coordinate system. Then, the $\left(\theta_{pitch},\theta_{yaw}\right)$ are computed as Equations (11) and (12):

(11) $$\theta_{pitch}=\arcsin\left(-y_{gn}\right)$$

(12) $$\theta_{yaw}=\arctan\frac{x_{gn}}{z_{gn}}$$

### 2.3. The Architecture of FG-Net

Based on the geometric knowledge mentioned above, we can ascertain that the task for the network is to learn the mapping *f* from the input images (face image I and eye image E) to 2D gaze angles $\left(\theta_{pitch},\theta_{yaw}\right)$ in the virtual camera coordinate system as Equation (13).

(13) $$\left(\theta_{pitch},\theta_{yaw}\right)=f\left(I,E\right)$$

**Figure 2:**
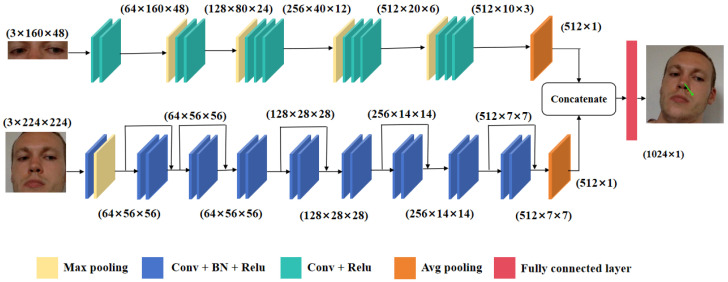
The architecture of FG-Net. It consists of two branches: the eye branch and the face branch. The inputs of FG-Net are paired eye and face images. The eye branch is a VGG-like network and the face branch is a ResNet-18 network.

The network architecture is shown in Figure 2. In order to extract features of the normalized images, we build a dual-branch network named FG-Net. For the face branch, we choose ResNet-18 as the backbone. It can solve the problem of gradient vanishing and gradient explosion in deep neural networks by introducing residual blocks. ResNet adds a shortcut connection that directly adds the input of the previous layer to the output of the later layer, allowing the gradient to be directly propagated to the previous layer. This structure not only increases the depth of the network but also improves its accuracy. The face branch consists of seven convolutional layers and five ResNet layers. The weights of convolutional layers and ResNet layers are pretrained on the ImageNet dataset. The input of the face branch is a 224 × 224 cropped facial image. The image first passes through a module composed of a 7 × 7 convolutional layer and a batch normalization (BN) layer, then is activated by the Relu function, and then noise is removed by a max pooling layer. Then, it passes through five ResNet layers and six 3 × 3 convolutional layers. The output size of the convolutional layers is 512 × 7 × 7. To reduce overfitting, we finally use an average pooling layer to reduce the number of features in the face branch. So, the final output of the face branch includes 512 features.

We adopt VGG-16 as the backbone for the eye branch to extract eye features. This is due to its small kernel size, which allows for a deeper network with fewer parameters and better nonlinear representation capability. The eye branch consists of thirteen convolutional layers and four maxpooling layers. The weights of convolutional layers are pretrained on the ImageNet dataset. Similarly, this branch also ends with an average pooling layer in order to speed up convergence and reduce the risk of overfitting. Finally, we concatenate the output from the two branches to combine extracted features and send them into the fully connected layer to predict the gaze vector in the normalized space.

### 2.4. 3D Gaze Estimation

The 3D gaze estimation aims to infer the 3D gaze vector $g_{p}$ = ($x_{gp}$, $y_{gp}$, $z_{gp}$) from 2D space gaze angle vector $\left(\theta_{pitch},\theta_{yaw}\right)$ and compute the angular difference between the estimated and ground truth 3D gaze vectors. When obtaining $\theta_{pitch}$ and $\theta_{yaw}$, we can compute the gaze vector $g_{p}$ by Equations (14)–(16):

(14) $$x_{gp}=-\frac{\cos(\theta_{pitch})}{\sin(\theta_{yaw})}$$

(15) $$y_{gp}=\sin\left(\theta_{pitch}\right)$$

(16) $$z_{gp}=-\frac{\cos(\theta_{pitch})}{\cos(\theta_{yaw})}$$

then, we obtain the gaze prediction $g_{p}$ = ($x_{gp}$, $y_{gp}$, $z_{gp}$) in the virtual camera coordinate system. Therefore, the angle between $g_{n}$ and $g_{p}$ can be computed as Equation (17):

(17) $$\langle g_{n},g_{p}\rangle =\arccos\frac{x_{gn}x_{gp}+y_{gn}y_{gp}+z_{gn}z_{gp}}{\sqrt{\left(x_{gn}^{2}+y_{gn}^{2}+z_{gn}^{2}\right)\left(x_{gp}^{2}+y_{gp}^{2}+z_{gp}^{2}\right)}}$$

The lower angle between $g_{n}$ and $g_{p}$ corresponds to higher accuracy. Then, we convert the angle from the virtual camera coordinate system into the original camera coordinate system. According to Equation (9), we can obtain

(18) $$g_{o}=R_{n}^{-1}\cdot g_{n}$$

where $g_{o}$ is the ground truth gaze vector in the camera coordinate system.

The angle $\langle R_{n}^{-1}g_{n},R_{n}^{-1}g_{p}\rangle$ is the predicted angle between the ground truth gaze vector and the predicted gaze vector in the original camera space. In this study, we prove that there is no need to convert the gaze vector into the original camera coordinate system. The proof is presented as follows.

Note that the rotation matrix $R_{n}$ between the camera coordinate system and the virtual camera coordinate system is an orthogonal matrix, so the inverse matrix $R_{n}^{-1}$ is also an orthogonal matrix.

Assuming that $R_{n}^{-1}$ is shown as in Equation (19):

(19) $$R_{n}^{-1}=\left[\begin{array}{ccc} r_{11} & r_{12} & r_{13}\\ r_{21} & r_{22} & r_{23}\\ r_{31} & r_{32} & r_{33} \end{array}\right]$$

then, we can compute the ground truth gaze vector $g_{o}$ and the prediction $g_{p}^{{}^{\prime }}$ in the original camera coordinate system using Equations (18) and (20):

(20) $$g_{p}^{{}^{\prime }}=R_{n}^{-1}\cdot g_{p}$$

so we obtain $g_{o}$ and $g_{p}^{{}^{\prime }}$:

(21) $$g_{o}=\left[\begin{array}{c} r_{11}x_{gn}+r_{12}y_{gn}+r_{13}z_{gn}\\ r_{21}x_{gn}+r_{22}y_{gn}+r_{23}z_{gn}\\ r_{31}x_{gn}+r_{32}y_{gn}+r_{33}z_{gn} \end{array}\right]$$

(22) $$g_{p}^{{}^{\prime }}=\left[\begin{array}{c} r_{11}x_{gp}+r_{12}y_{gp}+r_{13}z_{gp}\\ r_{21}x_{gp}+r_{22}y_{gp}+r_{23}z_{gp}\\ r_{31}x_{gp}+r_{32}y_{gp}+r_{33}z_{gp} \end{array}\right]$$

the angle between $g_{o}$ and $g_{p}^{{}^{\prime }}$ can be calculated by Equation (23):

(23) $$\langle g_{o},g_{p}^{{}^{\prime }}\rangle =\arccos\frac{g_{o}\cdot g_{p}^{{}^{\prime }}}{\left|\right|g_{o}\left|||\right|g_{p}^{{}^{\prime }}\left|\right|}$$

where $g_{o}\cdot g_{p}^{{}^{\prime }}$ is calculated as Equation (24):

(24) $$\begin{array}{c} g_{o}\cdot g_{p}^{{}^{\prime }}=\left(r_{11}x_{gn}+r_{12}y_{gn}+r_{13}z_{gn}\right)\left(r_{11}x_{gp}+r_{12}y_{gp}+r_{13}z_{gp}\right)\\ +\left(r_{21}x_{gn}+r_{22}y_{gn}+r_{23}z_{gn}\right)\left(r_{21}x_{gp}+r_{22}y_{gp}+r_{23}z_{gp}\right)\\ +\left(r_{31}x_{gn}+r_{32}y_{gn}+r_{33}z_{gn}\right)\left(r_{31}x_{gp}+r_{32}y_{gp}+r_{33}z_{gp}\right) \end{array}$$

and $\left|\right|g_{o}\left|\right|$ and $\left|\right|g_{p}^{{}^{\prime }}\left|\right|$ are computed as Equation (25) and Equation (26), respectively:

(25) $$\left|\right|g_{o}\left|\right|=\sqrt{\begin{array}{c} {\left(r_{11}x_{gn}+r_{12}y_{gn}+r_{13}z_{gn}\right)}^{2}+{\left(r_{21}x_{gn}+r_{22}y_{gn}+r_{23}z_{gn}\right)}^{2}+{\left(r_{31}x_{gn}+r_{32}y_{gn}+r_{33}z_{gn}\right)}^{2} \end{array}}$$

(26) $$\left|\right|g_{p}^{{}^{\prime }}\left|\right|=\sqrt{\begin{array}{c} {\left(r_{11}x_{gp}+r_{12}y_{gp}+r_{13}z_{gp}\right)}^{2}+{\left(r_{21}x_{gp}+r_{22}y_{gp}+r_{23}z_{gp}\right)}^{2}+{\left(r_{31}x_{gp}+r_{32}y_{gp}+r_{33}z_{gp}\right)}^{2} \end{array}}$$

$R_{n}^{-1}$ is an orthogonal matrix, so each vector in it has a magnitude of one, and the column vectors are mutually orthogonal. They are presented as Equation (27):

(27) $$\sum_{i=1}^{3}r_{ij}r_{ik}=\left\{\begin{array}{cc} 0 & ,\;j\neq k\\ 1 & ,\;j=k \end{array}\right.$$

where *j* and *k* take the values 1, 2, and 3.

Therefore, expanding Equations (24)–(26) and combining like terms, and substituting Equation (27) into them, we can obtain the result as shown in Equations (28)–(30):

(28) $$g_{o}\cdot g_{p}^{{}^{\prime }}=x_{gn}x_{gp}+y_{gn}y_{gp}+z_{gn}z_{gp}$$

(29) $$\left|\right|g_{o}\left|\right|=\sqrt{x_{gn}^{2}+y_{gn}^{2}+z_{gn}^{2}}$$

(30) $$\left|\right|g_{p}^{{}^{\prime }}\left|\right|=\sqrt{x_{gp}^{2}+y_{gp}^{2}+z_{gp}^{2}}$$

substituting Equations (28)–(30) into Equation (23), we can obtain

(31) $$\langle g_{o},g_{p}^{{}^{\prime }}\rangle =\arccos\frac{x_{gn}x_{gp}+y_{gn}y_{gp}+z_{gn}z_{gp}}{\sqrt{\left(x_{gn}^{2}+y_{gn}^{2}+z_{gn}^{2}\right)\left(x_{gp}^{2}+y_{gp}^{2}+z_{gp}^{2}\right)}}$$

and it is equal to $\langle g_{n},g_{p}\rangle$. In this condition, we demonstrate that the angle between the ground truth of gaze vector and the prediction of gaze vector remains unchanged when using orthogonal matrices for coordinate transformation. This eliminates the need to convert the gaze vectors in the normalized coordinate system to the original coordinate system, thus improving accuracy and saving computational resources.

## 3. Experiments and Results Analysis

To validate the effectiveness of the framework FreeGaze, we performed a series of experiments on two publicly available datasets: MPIIGaze [18] and EyeDiap [19]. Initially, we performed ten-fold cross-validation to showcase the fundamental performance of the proposed framework of 3D gaze estimation. Subsequently, we conducted ablation studies to investigate the impact of employing different facial regions for normalization on gaze estimation. Finally, we carried out ablation experiments to assess the contribution of each branch in the proposed FG-Net.

### 3.1. Datasets and Preprocessing

**Figure 3:**
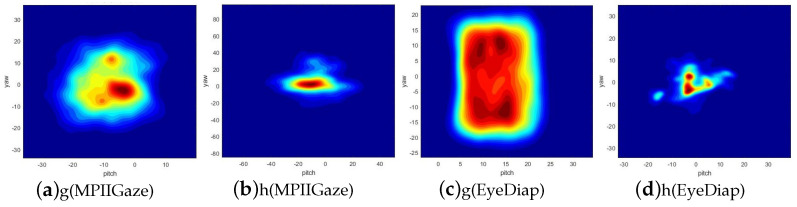
Distributions of gaze angle (g) and head pose (h) on the MPIIGaze and EyeDiap datasets in the normalized space.

There are a total of 15 participants in the MPIIGaze dataset, and its gaze target is screen targets. It consists of a large collection of images taken from different angles, capturing the faces of individuals in various settings. The images in the MPIIGaze dataset cover a wide range of variations, such as variations in appearances and head poses. These variations make it a challenging dataset for evaluating gaze estimation algorithms in realistic scenarios. Figure 3a,b summarize the distributions of its gaze angles and head poses in the normalized space. For gaze angles, the pitch angles are within the range of [−36.25°,19.97°], and the yaw angles are within the range of [−34.16°,36.49°]. For head poses, the pitch angles are within the range of [−76.17°,50.99°], and the yaw angles are within the range of [−85.36°,97.02°]. For the MPIIGaze dataset, we used the facial center provided in the dataset as the starting point for the 3D gaze vector. We used the center of the detected face landmarks as the point faced to the normalized camera. After normalization, we obtained normalized face images of 224 × 224 pixels, and then we cropped the face images to obtain eye images of 160 × 48 pixels. Finally, we normalized the pixel values of the RGB images to between 0 and 1. If this is not completed, the gradient transmitted to the input layer during backpropagation will become very large, which is not conducive to model convergence.

The other dataset, EyeDiap, has a total of 16 participants and 3-minute videos for each subject. Its gaze target includes screen targets and floating targets. Moreover, the videos can be further divided into static and moving head pose for each subject. To maintain consistency with previous studies and facilitate comparison, we only used the screen targets for evaluation and took one image every five frames from four VGA videos of each participant provided in this dataset. Figure 3c,d summarize the distributions of the gaze angles and head poses in the normalized space. For gaze angles, the pitch angles are within the range of [−3.91°,34.49°], and the yaw angles are within the range of [−26.69°,22.94°]. For head poses, the pitch angles are within the range of [−32.61°,39.40°], and the yaw angles are within the range of [−34.56°,34.92°]. We filtered out frames that met at least one of the following conditions: (1) the face of the participant is not detected; (2) the annotation is not available. We used two iris centers provided in the dataset to calculate the midpoint as the starting point of the 3D gaze vector. Similarly, we took the center of the detected face landmarks as the facing point of the normalized camera and implemented the same normalization step on EyeDiap as on MPIIGaze. We unified all coordinates into the camera coordinate system before data normalization in both datasets.

### 3.2. Implementation Details

The normalized datasets are divided into ten folds, and the models are trained by cross-validation. Nine subsets are used as the training set, and the remaining one is the validation set.

We trained the network on Pytorch-1.40 using a NVIDIA GeForce GTX-1660 GPU. We chose Adam as the optimizer with an initial learning rate of 0.001, and with default momentum values $\beta_{1}=0.9$ and $\beta_{2}=0.999$. The learning rate *ℓ* decays as Equation (32):

(32) $$\ell =\ell_{initial}\times \gamma \times s$$

where γ represents the decay rate with a value of 0.8, and s represents the period of learning rate decay, which is set to 1. We use the difference between the predicted and ground-truth 2D gaze angle vectors as loss function.

### 3.3. Ten-Fold Cross-Validation Evaluation

In order to illustrate the basic performance of our proposed framework FreeGaze, we compared it with other state-of-the-art methods on the MPIIGaze and EyeDiap datasets. The comparison results are listed in Table 2.

The 3D angular error refers to the angular difference between ground truth and prediction. From Table 1, we can see that FreeGaze achieves the best results on both the MPIIGaze and EyeDiap datasets. The MPIIGaze dataset covers significant variation in appearances. From the result, we can see that FreeGaze can guarantee high accuracy against various appearance challenges.

Meanwhile, we can see that our proposed method also ranks best on gaze estimation accuracy on the EyeDiap dataset. The proposed framework FreeGaze has a significant improvement on the EyeDiap dataset compared with other state-of-the-art methods. We believe that this can be attributed to the landmark detection and the improved normalization technique. The automatic landmark detection method enables more accurate detection of facial landmarks, which in turn allows for more precise calculation of head pose and eliminates the need for laborious manual annotation. The improved normalization technique eliminates the need to convert the network’s results from the normalized space to the original space, allowing us to directly calculate the error in the normalized space, which is more straightforward and efficient.

### 3.4. Ablation Studies

#### 3.4.1. The Effectiveness of Facial Landmarks in Different Facial Regions for Normalization

**Figure 4:**
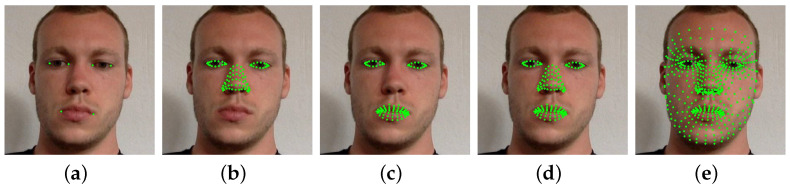
Facial landmarks in different facial regions used for normalization. (**a**) Eye and mouth corners; (**b**) eyes and nose region; (**c**) eyes and mouth region; (**d**) eyes, nose, and mouth region; (**e**) full face region.

Gaze direction is not only related to the eyes’ appearance cues but also to the orientation of the face. The accuracy of head pose estimation is crucial in achieving accurate gaze estimation. Therefore, we introduced the hypothesis that using facial landmarks of different facial regions for normalization would have an impact on gaze estimation accuracy. Previous studies [14,18] annotated face images with six facial landmarks, which are eye and mouth corners, as shown in Figure 4a. Most other researchers also adopt the same approach. However, this method does not provide the impact of normalization results on gaze estimation accuracy. Therefore, we employed a state-of-the-art method that can detect 468 facial landmarks [33], as shown in Figure 4e, enabling us to normalize images using landmarks of different facial regions, as shown in Figure 4. Moreover, this process eliminates the manual annotation of facial landmarks and allows for the flexible selection of appropriate facial landmarks. To study the impact of using facial landmarks in different facial regions for normalization on the accuracy of gaze estimation, we conducted five sets of experiments. We used landmarks from the five following regions for normalization: (1) eye and mouth corners; (2) eyes and nose; (3) eyes and mouth; (4) eyes, nose, and mouth; and (5) full face.

The preprocessing results are sent to the FG-Net we have built for accuracy evaluation. Then, we perform ablation experiments on the above-mentioned datasets. Table 3 shows the experimental results on the MPIIGaze and EyeDiap datasets.

For the MPIIGaze dataset, our experiments show that the use of different facial regions for preprocessing does impact the accuracy of gaze prediction. Only using corners of the eyes and mouth is insufficient to accurately estimate head pose, resulting in lower accuracy. Using eyes and nose region for preprocessing can improve the accuracy by 0.15° due to the close relationship between the nose and head pose. However, the improvement in accuracy using eyes and mouth region is only 0.04° compared to using the corners of the eyes and mouth, mainly because the mouth corners represent major information conveyed by the mouth. There is also synergy between the mouth and nose regions, leading to 0.2° improvement in accuracy when combined with the eyes region. Landmarks of the full face may be occluded when the face is tilted, resulting in similar effects between using the entire face and using the eyes and nose region.

For the EyeDiap dataset, the use of different facial regions for preprocessing has minimal impact on the accuracy of gaze estimation. This could be due to the low image quality of the dataset. The image resolution in the MPIIGaze dataset is 1280 × 720, while, in the EyeDiap dataset, it is 640 × 480. Due to the low image quality, there are fewer details, resulting in less impact on gaze estimation when using different facial regions for normalization. In these cases, using only six landmark points from the corners of the eyes and mouth for normalization is sufficient.

The MPIIGaze dataset has good image quality, while the EyeDiap dataset has lower image quality. From the above results, it can be observed that, in cases of higher image quality, using more facial landmarks for preprocessing can lead to higher accuracy. However, when the image quality is lower, using the six landmarks of the corners of the eyes and mouth is sufficient.

#### 3.4.2. The Effectiveness of Dual-Branch Architecture

In order to study the contribution of each branch in FG-Net to the gaze estimation, we split the network into two separate branches, eye branch and face branch. We conducted experiments with eye branch only or face branch only to estimate 3D gaze. To maintain consistency, we used the preprocessing data obtained with 468 facial landmarks and 166 facial landmarks for evaluation, respectively.

Table 4 shows the ablation results on the MPIIGaze and EyeDiap datasets. From the results of the MPIIGaze dataset, we find that the eye branch makes little contribution to gaze estimation, while the face branch plays a significant role. This could be attributed to the dataset’s wide range of head poses, which in turn results in insufficient information from eye images to accurately estimate gaze direction. By contrast, the full face provides not only eye features but also head pose information, leading to improved accuracy compared to using only the eye branch. Despite combining the eye and face branches, there was only a marginal improvement in accuracy, which may be attributed to either feature overlap or the eye branch not contributing significantly.

In contrast to the MPIIGaze dataset, the results on the EyeDiap dataset indicate that the eye branch plays a significant role in gaze estimation. The eye branch achieves a relatively high accuracy, with an error of 0.15° less than the face branch. This variation can be attributed to the low image quality in the EyeDiap dataset compared to MPIIGaze. Another reason could be that the EyeDiap dataset encompasses a smaller range of head poses compared to the MPIIGaze dataset, meaning that the cropped eye images from the EyeDiap dataset contain main information for gaze estimation. Similarly, the combination of the two branches has little improvement in accuracy of gaze estimation due to feature overlap.

From the results above, we can conclude that the final estimation accuracy is mainly dependent on the face branch network, and the eye branch also contributes a bit. We should adaptively select whether to estimate gaze direction using the eyes or the face based on the quality of the image. When the image quality is high, using facial features rather than solely relying on eye features can lead to better gaze estimation performance. Additionally, a dual-branch CNN can contribute to improving accuracy a bit. When the image quality is low, using only facial features or eye features separately, or even combining facial and eye features, does not have a significant impact on the results. It is reasonable to estimate gaze using facial features when there is a wide range of head poses because facial features not only include eye appearances but also encompass head pose information. When using images normalized with 168 facial landmarks, it is possible to achieve a similar effect as images normalized with 468 facial landmarks. This can help reduce computational load.

#### 3.4.3. The Effectiveness of the Improved Normalization Method

In order to demonstrate the basic performance of the improved normalization method, we conducted comparative experiments on MPIIGaze and EyeDiap datasets to compare the performance of the improved normalization method and the original normalization method in gaze estimation accuracy and computational time. The computational time represents the time required to estimate the predicted value of an image. The comparison results are listed in Table 5.

From the results, we can see that the improved normalization method has much higher accuracy than the original normalization method, and it also improves performance in terms of computational time. For the MPIIGaze dataset, the improved normalization method improves accuracy by 4.89° and improves performance by 11.74% in time. For the EyeDiap dataset, the improved normalization method improves accuracy by 2.83° and improves performance by 8.61% in time.

Compared to the performance on the EyeDiap dataset, there is a more performance improvement on the MPIIGaze dataset. We believe the improvement between the original normalization method and the improved normalization method is relevant to data distribution. As shown in Figure 3, the MPIIGaze dataset has a wider range of gaze angle and head pose distribution than EyeDiap; in this condition, the intermediate steps make the error superposition larger when using the original normalization method. When using the original normalization method, the 3D angular error between the ground truth and the estimation of gaze vectors in the normalized coordinate system is different from in the original camera coordinate system, so we need to convert the estimations of gaze vectors from the normalized coordinate system to the original camera coordinate system. As described in Equation (7), M is not an orthogonal matrix, so, as a result, when conducting the converting, the angle error between two vectors will be amplified. When using an improved normalization method, the estimations of 3D gaze vectors do not need to be converted to the original camera coordinate system, and the 3D angular error in the normalized coordinate system is the same as that in the original camera coordinate system. This leads to a significant effect on the accuracy of 3D gaze estimation. The use of improved normalization methods can avoid unnecessary intermediate step calculations, thereby reducing computational time.

## 4. Conclusions

In this paper, we develop a framework named FreeGaze to estimate gaze in facial videos. We use a new method to detect landmarks and analyze the influence of using landmarks from different facial regions for data preprocessing on gaze estimation accuracy, which provides a basis for reducing the computational cost of the entire system. We propose a dual-branch CNN, named FG-Net, and conduct ablation experiments on both the MPIIGaze and EyeDiap datasets to study the contributions of the eye region and full face to gaze estimation, providing experience for reducing the network size in the future. We conduct comparative experiments to show the advantage of the improved normalization method. For MPIIGaze, the improved normalization method improves accuracy by 4.89° and improves performance by 11.74% in time, and, for EyeDiap, the values are 2.83° and 8.61%. Our experimental results show that our method achieves state-of-the-art accuracy on both datasets. In future work, we will apply an attention mechanism in gaze estimation to improve the performance in extreme angle environments. We will make effective improvements in feature extraction and fusion to improve the performance metrics, especially in eye and face features.

## Figures and Tables

**Table 1:** Comparison with other state-of-the-art methods in features (eye and face) and advantages.

Method	Eye	Face	Advantages
Multimodal CNN [18]	y	n	Low complexity
Gazemap [20]	y	n	Robustness to head pose and image quality
Multiview CNN [21]	y	n	Multitask solution
Differential NN [22]	y	n	Less calibration
DRNet [23]	y	n	Robustness to noise
U-Train [24]	y	n	Unsupervised
Spatial weights CNN [16]	n	y	Robustness to facial appearance variation
BPA-Net [25]	n	y	Robustness to facial appearance variation
Recurrent CNN [17]	n	y	Temporal modality
DEA-Net [26]	y	n	Less samples
iTracker [27]	y	y	High generalization in different datasets
Bi-LSTM [28]	y	y	Low complexity and robustness to resolution
Gaze360 [29]	n	y	High generalization in real scene
GEDD-Net [30]	y	y	low complexity high performance calibration
STTDN [31]	y	y	feature fusion and dynamic feature extraction
FreeGaze (Ours)	y	y	Improved normalization method and landmarks’ impact on gaze estimation

**Table 2:** Comparison with other state-of-the-art methods on MPIIGaze and EyeDiap.

Method	3D Angular Error (°)	
	MPIIGaze	EyeDiap
Multimodal CNN [18]	6.3	-
Spatial weights CNN [16]	4.8	6.0
Dilated-Convolutions [35]	4.8	-
Recurrent CNN [17]	-	3.4
L2CS-Net [36]	3.92	-
Bi-LSTM [28]	4.18	5.84
CA-Net [37]	4.1	5.3
FARE-Net [38]	4.3	5.71
DEA-Net [26]	4.38	-
GEDD-Net [30]	4.5	5.4
STTDN [31]	3.73	5.02
U-Train [24]	-	6.79
DRNet [23]	4.57	6.14
FreeGaze	3.11	2.75

**Table 3:** Ablation studies on the effectiveness of different facial regions for normalization on MPIIGaze and EyeDiap.

Facial Regions for Preprocessing	Number of Landmarks	3D Angular Error (°)	
		MPIIGaze	EyeDiap
Corners of eyes and mouth	6	3.26	2.79
Eyes and nose	92	3.11	2.79
Eyes and mouth	112	3.22	2.80
Eyes, nose, and mouth	166	3.06	2.78
Full face	468	3.11	2.75

**Table 4:** Ablation studies of FG-Net on MPIIGaze and EyeDiap.

Number of Landmarks (Facial Regions)	Branches	3D Angular Error (°)	
		MPIIGaze	EyeDiap
468 (full face)	Eye branch	6.33	2.88
	Face branch	3.13	2.73
	Dual branch	3.11	2.75
166 (eye, nose, and mouth)	Eye branch	6.39	2.90
	Face branch	3.13	2.76
	Dual branch	3.06	2.78

**Table 5:** Ablation studies on the improved normalization method for MPIIGaze and EyeDiap.

Normalization Method	3D Angular Error (°)		Computational Time (ms)	
	MPIIGaze	EyeDiap	MPIIGaze	EyeDiap
Original	8.00	5.58	5.96	5.11
Improved	3.11	2.75	5.26	4.67

## Data Availability

The data that support the findings of this study are openly available at https://www.perceptualui.org/research/datasets/MPIIFaceGaze/ (accessed on 1 May 2023) and https://www.idiap.ch/en/dataset/eyediap (accessed on 1 May 2023).
